# *Arabidopsis EMB1990* Encoding a Plastid-Targeted YlmG Protein Is Required for Chloroplast Biogenesis and Embryo Development

**DOI:** 10.3389/fpls.2018.00181

**Published:** 2018-02-16

**Authors:** Hongyu Chen, Shuqin Li, Lu Li, Hengjin Hu, Jie Zhao

**Affiliations:** State Key Laboratory of Hybrid Rice, College of Life Sciences, Wuhan University, Wuhan, China

**Keywords:** *Arabidopsis*, chloroplast, embryo development, plastid gene expression, YLMG

## Abstract

In higher plants, embryo development originated from fertilized egg cell is the first step of the life cycle. The chloroplast participates in many essential metabolic pathways, and its function is highly associated with embryo development. However, the mechanisms and relevant genetic components by which the chloroplast functions in embryogenesis are largely uncharacterized. In this paper, we describe the *Arabidopsis EMB1990* gene, encoding a plastid-targeted YlmG protein which is required for chloroplast biogenesis and embryo development. Loss of the *EMB1990/YLMG1-1* resulted in albino seeds containing abortive embryos, and the morphological development of homozygous *emb1990* embryos was disrupted after the globular stage. Our results showed that *EMB1990/YLMG1-1* was expressed in the primordia and adaxial region of cotyledon during embryogenesis, and the encoded protein was targeted to the chloroplast. TEM observation of cellular ultrastructure showed that chloroplast biogenesis was impaired in *emb1990* embryo cells. Expression of certain plastid genes was also affected in the loss-of-function mutants, including genes encoding core protein complex subunits located in the thylakoid membrane. Moreover, the tissue-specific genes of embryo development were misexpressed in *emb1990* mutant, including genes known to delineate cell fate decisions in the SAM (shoot apical meristem), cotyledon and hypophysis. Taken together, we propose that the nuclear-encoded YLMG1-1 is targeted to the chloroplast and required for normal plastid gene expression. Hence, YLMG1-1 plays a critical role in *Arabidopsis* embryogenesis through participating in chloroplast biogenesis.

## Introduction

The individual life of flowering plant begins with the fertilized egg cell, which gives rise to the mature embryo through a series of programmed cell divisions and differentiations. In *Arabidopsis*, embryo development follows a regular and predictable pattern, and this precisely controlled process is regulated by a complicated network of gene expression (Boscá et al., 2011; Luo et al., 2014; Crawford et al., 2015; ten Hove et al., 2015; Ueda et al., 2017). Previous studies predicted that about 1000 genes are required for the *Arabidopsis* embryogenesis, and a portion of these genes have been identified and characterized as essential through the isolation of embryo-defective (*emb*) mutants (Meinke et al., 2008; Lloyd and Meinke, 2012). Functional analysis showed that most of the *EMB* genes function in basic cellular processes such as DNA, RNA, and protein synthesis, which are likely to have counterparts with essential genes in yeast and *Caenorhabditis elegans* (Tzafrir et al., 2004; Devic, 2008). The identification and functional characterization of more essential genes in *Arabidopsis* can thus help us develop a deeper understanding of the molecular mechanisms and regulatory networks underlying embryogenesis in eukaryotes.

In higher plants, the chloroplast not only performs photosynthesis but also synthesizes amino acids, lipids and phytohormones; and so it plays a central role in metabolism, growth and differentiation of plant cells (Inaba and Ito-Inaba, 2010). According to the theory of endosymbiosis, the chloroplast in higher plants descended from a photosynthetic prokaryote engulfed by the primitive eukaryotic ancestor of land plants (Cavalier-Smith, 2004; Reyes-Prieto et al., 2007). As a result, chloroplasts contain their own genomes and gene expression systems. Although most of the prokaryotic genes were lost or transferred to the nuclear genome during the long evolution, modern chloroplasts clearly reflect their prokaryotic origins through their metabolic activities, genetic mechanisms, and protein transport complexes (Keeling, 2010; Pfalz and Pfannschmidt, 2015). Previous computational methods predicted that there are about 2000–4000 different proteins in the *Arabidopsis* chloroplast, and over 1000 chloroplast proteins have been experimentally verified (Friso et al., 2004; Heazlewood et al., 2007). The *Arabidopsis* chloroplast genome contains only 45 RNA-coding and 87 protein-coding genes, therefore the vast majority of chloroplast proteins are encoded by the nuclear genome, synthesized in the cytoplasm and imported into the chloroplast. For example, nearly all the protein complexes in chloroplasts are composed of a mixture of nucleus- and chloroplast-encoded subunits, and the NEP (nucleus-encoded polymerase) initiates transcription of a set of chloroplast genes involved in transcription and protein synthesis (Shiina et al., 2005). Chloroplast biogenesis is primarily controlled by nuclear genes, whereas signaling molecules can be generated in the chloroplast, and transduced into the nucleus to influence expression of nuclear genes (Petrillo et al., 2014; Chan et al., 2016; Ren et al., 2017). The coordination of chloroplast and nuclear gene expression is integrated through intracellular signaling, and required for chloroplast biogenesis as well as growth and development of higher plants.

In *Arabidopsis* embryo cells, chloroplast biogenesis is initiated when embryo development reaches the late globular stage (Mansfield and Briarty, 1991). The differentiation from proplastid to chloroplast requires abundant nuclear genes, and mutations in these genes often interfere with chloroplast functions, leading to embryo lethality. A comprehensive dataset of 381 nuclear genes encoding chloroplast-localized proteins was established in *Arabidopsis*, among them there were 119 genes associated with an embryo-defective phenotype, indicating that the function of the chloroplast is essential for *Arabidopsis* embryogenesis (Bryant et al., 2011; Lloyd and Meinke, 2012). Furthermore, several studies have confirmed that dysfunction of chloroplast-related genes typically leads to abnormal embryogenesis, with arrested embryo development mainly occurring at or after the globular stage (Yin et al., 2012; Lu et al., 2014; Chen et al., 2015; Ke et al., 2017), or in some cases after the heart stage (Liang et al., 2010; Feng et al., 2014). However, there are also many chloroplast-related mutations that do not affect embryo morphogenesis but result in albino embryos and seedlings (Pyo et al., 2013; Kremnev and Strand, 2014). Recently, light-induced chloroplast biogenesis was found to be unnecessary for embryo morphogenesis, but was strongly required for the accumulation of storage reserves during embryo maturation in *Arabidopsis* (Liu et al., 2017). All of these studies demonstrated the critical role of chloroplasts in embryo development, and the diverse chloroplast-related genes participated in different aspects of chloroplast biogenesis, thus functioning in different stages of *Arabidopsis* embryogenesis.

To better understand the functional mechanisms by which chloroplasts participate in *Arabidopsis* embryogenesis, based on gene annotations and predicated function in the SeedGenes Database (Meinke et al., 2008), we searched for the *EMB* genes encoding putative plastid-targeted proteins, then T-DNA insertion mutants of these genes were obtained from the Arabidopsis Biological Resource Center. One of these genes named *EMB1990* (At3g07430) was found to be required for the proper distribution of nucleoids in chloroplast (Kabeya et al., 2010), but its function in embryo development is unknown. Analysis of protein sequence showed that *EMB1990* was homologous to the bacterial gene *YlmG*. In the genome of *Arabidopsis thaliana*, four homologs of YlmG were identified (At3g07430, At4g27990, At5g21920, and At5g36120), the At3g07430 was named to be *AtYLMG1-1* (Kabeya et al., 2010). Here, we report a comprehensive functional characterization of *EMB1990*, which encodes the plastid-targeted AtYLMG1-1, in embryo development and chloroplast biogenesis. We found that the mutation of *AtYLMG1-1* led to seed abortion, which might due to the failure of chloroplast biogenesis during embryo development, and that several important genes known to delineate cell fate decisions were up-regulated or down-regulated in the *emb1990* abortive ovules. Therefore, we concluded that the plastid-targeted YLMG1-1 thus plays a crucial role in *Arabidopsis* embryo development through participation in chloroplast biogenesis.

## Materials and Methods

### Plant Materials and Growth Conditions

The two *Arabidopsis emb1990* alleles, *emb1990-1* (CS16162) and *emb1990-2* (CS24080) were obtained from the Arabidopsis Biological Resource Center^1^. Then the locations of T-DNA insertions in these two mutants were verified by PCR and sequencing. The *Arabidopsis* plants were cultivated in a greenhouse at Wuhan University at 22 ± 2°C with a 16-h light and 8-h dark cycle.

For dark treatment, *Arabidopsis* seeds were sterilized and plated on solid 1/2 MS medium. After stratified for 2 days at 4°C, the seeds were cultivated under the normal light conditions for 6 days at 22°C. And then 6 DAG (day after germination) seedlings were transferred to dark condition for 1 day, while the control tested seedlings grew under the normal light cycle. For the light recovery, treated seedlings were replaced under the normal light cycle for another 1 day, and the 8 DAG seedlings grew under the normal light cycle as the control.

### Ovule Clearing and Embryo Observation

Fresh ovules were removed from siliques under a stereomicroscope with forceps and insulin needles, then immersed in Hoyer’s solution (chloral hydrate: glycerol: water = 8: 1: 2, w/v/v) for minutes to hours depending on the embryo development stage (Berleth and Jürgens, 1993). After that the cleared ovules were observed by differential interference contrast under an inverted microscope Olympus TH4-200 (Olympus, Japan) equipped with a CCD of SPOT Digital Microscope Camera (Diagnostic Instruments).

### RNA Extraction and RT-PCR

Total RNA from different kinds of *Arabidopsis* tissues was isolated by RNAiso Plus, except the siliques and ovules were extracted using MiniBEST Plant RNA Extraction Kit (TaKaRa, Japan). Afterward, RNA was transcribed into cDNA using random hexamer primer with RevertAid RT Reverse Transcription Kit (Fermentas, United States), following the manufacturer’s instructions. Then the cDNA was used as template for subsequent PCR analysis with specific primers (Supplementary Table S1). Real-time PCR was performed using TransStart Top Green qPCR SuperMix (TransGen, China) in a BIO-RAD CFX Connect machine (BIO-RAD, United States). At least three independent biological replicates were made for real-time PCR analysis for different samples, and three technical replicates were taken in each biological replicate. The relative expression levels were analyzed by the comparative CT method (Schmittgen and Livak, 2008), and the *GAPDH* was applied as reference gene for quantitative PCR analysis. Statistical analysis was performed by Student’ *t*-test, and values of *P* were used to determine the statistically significant.

### *EMB1990/YLMG1-1* Genomic DNA Cloning and Mutant Complementation

The full-length sequence of *EMB1990/YLMG1-1* genomic DNA fragment, including the 943 bp gene sequence and 609 bp upstream sequence of the ATG codon, was amplified from wild-type genome by PCR using the KOD-Plus-Neo DNA polymerase (TOYOBO, Japan) with a pair of specific primers (Supplementary Table S1). Then obtained fragment was digested with *Pst* I and *Sac* I, inserted into pCAMBIA1300 plasmid (Cambia, Australia), and verified by sequencing. After that the complementation vector was transferred into *emb1990* mutants by the floral-dip method (Clough and Bent, 1998). Then the transgenic seeds were screened on hygromycin plates, positive transformants were identified by PCR, and used for subsequent phenotype analysis.

### *YLMG1-1* Promoter and GUS/GFP Fusion

The promoter fragment of *YLMG1-1* was amplified with genome specific primers (Supplementary Table S1), and cloned into pCAMBIA1381Xb (Cambia, Australia) with *Bam*H I and *Pst* I to generate the *pYLMG1-1::GUS.* For the construction of *pYLMG1-1::YLMG1-1-Venus*, full-length coding sequence of *EMB1990/YLMG1-1* was amplified from inflorescences cDNA, the PCR product was digested with *Bam*H I and inserted into the modified pCAMBIA1300 containing a *35S-Venus* fragment to generate the *p35S::YLMG1-1-Venus.* Then this vector was used to generate *pYLMG1-1::YLMG1-1-Venus* through replacing the 35S promoter by its native promoter, which was amplified from wild-type genome with specific primers (Supplementary Table S1), and digested with *Pae* I and *Bam*H I. After sequencing the constructed vectors were transformed into *Arabidopsis* plants by the method described above.

### GUS Staining Analysis

The homozygous T4 generation *pYLMG1-1::GUS* transgenic plants were used for GUS staining analysis, and the experimental procedure was performed as the previous report (Deng et al., 2014). At last the stained samples from different *Arabidopsis* tissues were observed under an Olympus SZX12 stereomicroscope (Olympus, Japan) and photographed by a digital camera (Cool SNAP, RS Photometric).

### Subcellular Localization

The *p35S-Venus*, *p35S::YLMG1-1-Venus* and *pYLMG1-1::YLMG1-1-Venus* transgenic plants were obtained when investigating the expression of *YLMG1-1* during embryogenesis. Further, these transgenic lines were used for subcellular localization analysis. Mesophyll protoplasts were prepared from the 4-week-old transformants, and then used to analyze the subcellular localization of YLMG1-1 under an Olympus FV1000 confocal microscope with filters for Venus (excitation, 514 nm; emission, 526–600 nm) and chlorophyll autofluorescence (excitation, 488 nm; emission, 650–675 nm). After that the Olympus confocal software was used to generate the merged images from the different channels.

### CLSM Observation

To analyze the expression pattern of *YLMG1-1* during embryo development, confocal laser scanning microscopy (CLSM) was applied to detect the fluorescent signal in the *pYLMG1-1::YLMG1-1-Venus* transgenic lines. Fresh embryos at different stages were isolated from ovules, mounted in 10% glycerol, and then observed and photographed under an Olympus FV1000 confocal microscope with filters for Venus (excitation, 514 nm; emission, 526–600 nm).

### Transmission Electron Microscopy

The 5 DAP ovules from wild-type and *emb1990-1/*+ plants were dissected, fixed, embedded, and sectioned as described by Qin and Zhao (2006). After that the prepared samples were observed and photographed under a transmission electronic microscope (JEOL JEM-1400plus). During this process, five embryo samples of WT and *emb1990-1* were observed under the microscope. More than ten cells in different locations of each embryo sample were carefully analyzed, and the most representative pictures were selected to display the chloroplast structure in WT and *emb1990-1* embryo cells.

## Results

### Loss of *EMB1990/YLMG1-1* Result in Seed Abortion

**FIGURE 1:**
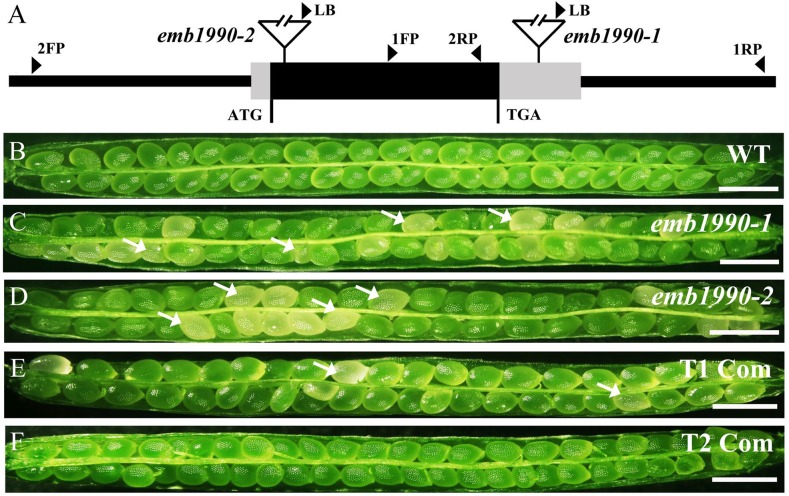
Characterization and complementation of *Arabidopsis emb1990* mutants. **(A)** Schematic diagrams of *EMB1990/YLMG1-1* gene. The positions of T-DNA insertions in *emb1990-1* and *emb1990-2* mutants are shown in the schematic diagrams. Arrowheads indicate the positions of primers used for genotyping. **(B–F)** Seed development in siliques of wild-type, mutants, and complemented plants. White arrows highlight the aborted white ovules, and the siliques were placed as morphological apical to basal from left to right. Bars = 1 mm.

*Arabidopsis EMB1990* was first identified to be essential for seed formation through the SeedGenes Project (Meinke et al., 2008). This gene was then found to be required for the proper distribution of nucleoid in chloroplast and named *AtYLMG1-1* on the basis of homology to the bacterial YlmG protein (Kabeya et al., 2010). To further investigate the biological function and mechanism of *EMB1990/YLMG1-1* in seed formation, two independent T-DNA insertion mutants of *EMB1990* were obtained from ABRC. After verifying the insertions by genomic PCR and sequencing, we found that the positions of the T-DNA insertions in *emb1990-1* (CS16162) and *emb1990-2* (CS24080) were located in the 3′UTR and within the only exon, respectively (**Figure 1A**).

Genotypic analysis of *emb1990-1* and *emb1990-2* progeny (*n* > 100 per line) showed that no homozygotes were produced by both mutants (Supplementary Figures S1A,B), and none of the heterozygous plants exhibited any vegetative developmental defects. Further phenotypic analysis revealed that a portion of ovules in the mature siliques were white: 24.6% in *emb1990-1*/+ and 25.9% in *emb1990-2*/+, compared with the frequency of 0.9% in wild type (**Figures 1B–D** and **Table 1**). The white ovules in *emb1990/*+ siliques then turned brown and wrinkled, occurring with the percentages close to 25%, the classical ratio of embryo-lethal mutants (Tzafrir et al., 2004). This finding indicated that null mutation of *EMB1990/YLMG1-1* leads to seed abortion in *Arabidopsis*.

**Table 1:** The percentage of abortive ovules in *emb1990-1* and *emb1990-2*.

Parental line	Normal ovules	Abortive ovules	% of abortive ovules	χ^2^
WT	1406	14	0.99%	/
*emb1990-1/*+	1205	394	24.64%	0.77
*emb1990-2/*+	1338	469	25.95%	0.42

To clarify whether a null mutation in *EMB1990/YLMG1-1* could result in gametophyte sterility, we performed further genetic analyses on the *emb1990*/+ lines. The T-DNA insertion in *emb1990-1*/+ mutant harbors the Basta (Bas) resistance gene, which facilitated segregation analysis of the mutant alleles. Progenies of self-pollinated *emb1990-1*/+ mutant segregate in a 2:1 ratio of Bas-resistant to Bas-sensitive (**Table 2**), the expected theoretical ratio for heterozygous to wild-type plants when the homozygous *emb1990-1* embryos were lethal (Meinke et al., 2008). We then performed reciprocal crosses with *emb1990-1*/+ to wild-type plants, and analyzed the segregation of Bas resistance in the progenies. The result showed that transmission efficiencies of both female and male gametophytes in *emb1990*/+ mutants were normal (**Table 2**), indicating that knock out of *EMB1990* did not affect the capacity of gametophytes.

**Table 2:** Genetic transmission analysis of *emb1990-1* in *Arabidopsis*.

Female × Male	BASTA^R^	BASTA^S^	BASTA^R^/BASTA^S^	TE (Female)	TE (Male)
*emb1990-1/*+ ×*emb1990-1/*+	1007	486	2.07:1	NA	NA
*emb1990-1/*+ × +*/*+	772	734	1.05:1	104.6%	NA
+*/*+ ×*emb1990-1/*+	693	708	0.98:1	NA	97.9%

*BASTA^R^, BASTA-resistant; BASTA^S^, BASTA-sensitive; TE, transmission efficiency = (BASTA^R^/BASTA^S^) × 100%; NA, not applicable.*

To confirm the seed abortion in *emb1990* mutants was caused by the disruption of *EMB1990*, genetic complementation was applied to test whether the white ovules could be rescued. A genomic fragment of *EMB1990/YLMG1-1*, including the 943 bp gene sequence and 609 bp upstream of the ATG codon, was introduced into both mutants. Then PCR screening and phenotypic analysis in the T2 progeny of the complementation lines showed that no aborted seeds were observed in homozygous *emb1990* mutants (**Figures 1E,F** and Supplementary Figures S1E,F). These results confirmed that *EMB1990/YLMG1-1* was responsible for the seed abortion in corresponding mutants, indicating the *EMB1990/YLMG1-1* gene is essential for seed formation in *Arabidopsis*.

### Morphological Development of Homozygous *emb1990* Embryo Is Disrupted after the Globular Stage

**FIGURE 2:**
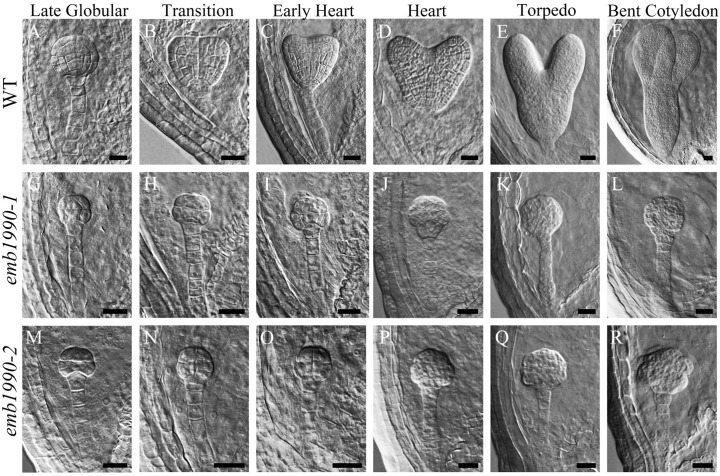
Embryo development in *emb1990-1* and *emb1990-2* mutants. **(A–F)** Embryos from late globular stage to bent cotyledon stage in wild-type ovules. **(G–L)** *emb1990-1* and **(M–R)** *emb1990-2* embryos from siliques at different development stage as similar as wild-type embryos showed in **(A–F)**. Scale bars = 20 μm.

To investigate the mechanisms of seed abortion in the *emb1990* heterozygous mutants, we examined ovule development in *emb1990-1*/+ and *emb1990-2*/+ mutants compared with wild-type plant, through a whole mount clearing technique. We found no obvious differences between wild-type and *emb1990*/+ embryos from the zygote up to the early globular stage (Supplementary Figure S2). Embryogenesis in wild-type continued to follow the programmed stages successively: globular, transition, heart, torpedo and curled cotyledon. Meanwhile in the white ovule of *emb1990*, embryo development was trapped at the globular stage (**Figure 2**), and the abnormal shape of the arrested *emb1990* embryo became more severe following the course of development.

When wild-type embryos reached the bent-cotyledon stage, the *emb1990* embryos were still unable to develop beyond the globular stage, and instead displayed abnormal cell division and shape alterations with no formation of shoot apical meristem or cotyledonous primordium (**Figures 2F,L,R**). These results indicate that morphological development of a homozygous *emb1990* embryo is disrupted after the globular stage, thus *Arabidopsis* embryogenesis requires the function of the *EMB1990/YLMG1-1* gene.

### The *EMB1990/YLMG1-1* Is Expressed Widely in a Variety of Tissues and Organs

**FIGURE 3:**
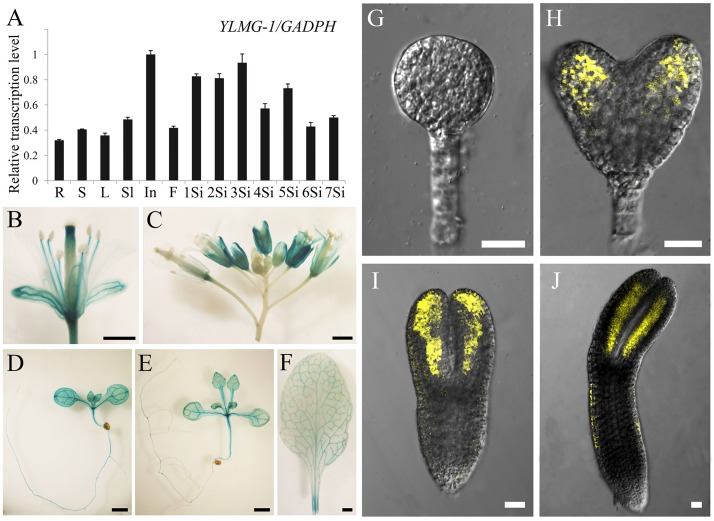
Expression pattern of *EMB1990/YLMG1-1* gene in *Arabidopsis* tissues and organs. **(A)** Expression levels of *EMB1990/YLMG1-1*genes in different tissues by qPCR assay. R, root; S, stem; L, leave; Sl, seedling; In, inflorescence; F, flower; 1Si, 1 DAP silique; 2Si, 2 DAP silique; 3Si, 3 DAP silique; 4Si, 4 DAP silique; 5Si, 5 DAP silique; 6Si, 6 DAP silique; 7Si, 7 DAP silique. **(B–F)** GUS activity in *pYLMG1-1::GUS* transgenic plants. **(B)** Flower; **(C)** inflorescence; **(D)** 7 DAG seedling; **(E)** 14 DAG seedling; **(F)** rosette leave. Scale bars = 2mm. **(G–J)** Fluorescence analysis of embryos at different stage from *pYLMG1-1::YLMG1-1-Venus* transgenic plants. **(G)** Globular stage; **(H)** Heart stage; **(G)** torpedo stage; **(G)** bent cotyledon stage. Scale bars = 20 μm.

To investigate the expression pattern of the *EMB1990/YLMG1-1* gene, quantitative PCR was performed to evaluate its relative transcription levels in various *Arabidopsis* tissues, with *GAPDH* applied as the reference gene. The results showed that *EMB1990/YLMG1-1* was expressed at different levels in nearly all examined tissues, including the vegetative and reproductive organs. The relative expression levels were most abundant in inflorescences and siliques, whereas the lowest transcript expression was detected in mature root, stem and leaf tissue (**Figure 3A**).

To further analyze the spatial expression pattern, the *EMB1990/YLMG1-1* promoter was fused with a β-glucuronidase (GUS) reporter to monitor its expression in *Arabidopsis* transgenic plants (*pYLMG1-1::GUS*). In the open flower, strong GUS signals were detected in sepals, filaments and stigmas, while the young flower bud in the inflorescence showed little signal compared to the flower after fertilization (**Figures 3B,C**). In 7DAG and 14DAG seedlings, the GUS staining was clearly observed in the shoot meristem, hypocotyl, root and vascular bundles of cotyledons, as well as in the veins of mature leaves (**Figures 3D–F**). In addition, we generated *pYLMG1-1::YLMG1-1-Venus* transgenic plants to evaluate the expression during embryo development. Fluorescence observation showed that no Venus signal could be detected at the early globular stage. After that stage, the Venus fluorescence was primarily observed in the cotyledon primordia of embryos in the heart stage, especially distributed on the paraxial side of embryo cotyledon primordia at the torpedo and bent cotyledon stage (**Figures 3G–J**). This expression pattern of *EMB1990/YLMG1-1* during embryo development was associated with the defective embryo phenotype after the globular stage in *emb1990* mutants.

### EMB1990/AtYLMG1-1 Is Localized in the Chloroplast

The SUBA database (The Subcellular Localization of Proteins in Arabidopsis Database^2^) was used for the prediction of EMB1990/YLMG1-1 exclusive targeting to the plastid (Hooper et al., 2017). Work by Kabeya et al., 2010 also revealed that EMB1990/YLMG1-1 localizes in small puncta on thylakoid membranes by immunofluorescence and immunoblot analysis. To confirm this localization, we generated a transgenic plant carrying a fusion of the EMB1990/YLMG1-1 coding sequence with the YFP variant Venus, under the control of a 35S promoter. Mesophyll protoplasts isolated from the *35S::Venus* and *35S::AtYLMG1-1-Venus* transgenic lines were observed under confocal microscopy to detect subcellular signals. The fluorescence images showed that AtYLMG1-1-Venus signals entirely overlapped with chlorophyll autofluorescence and were not localized in other places (Supplementary Figures S3D–F), while the control Venus signals were mainly distributed ubiquitously in the cell cytoplasm (Supplementary Figures S3A–C). To avoid interference from the strong 35S promoter, we also expressed the YLMG1-1-Venus under the native *YLMG1-1* promoter. Fluorescence in mesophyll protoplasts of *pYLMG1-1::YLMG1-1-Venus* confirmed the chloroplast localization of EMB1990/YLMG1-1 in *Arabidopsis* (Supplementary Figures S3G–I).

### The Expression of *EMB1990/YLMG1-1* Is Down-regulated by Dark and Up-regulated by Re-light

**FIGURE 4:**
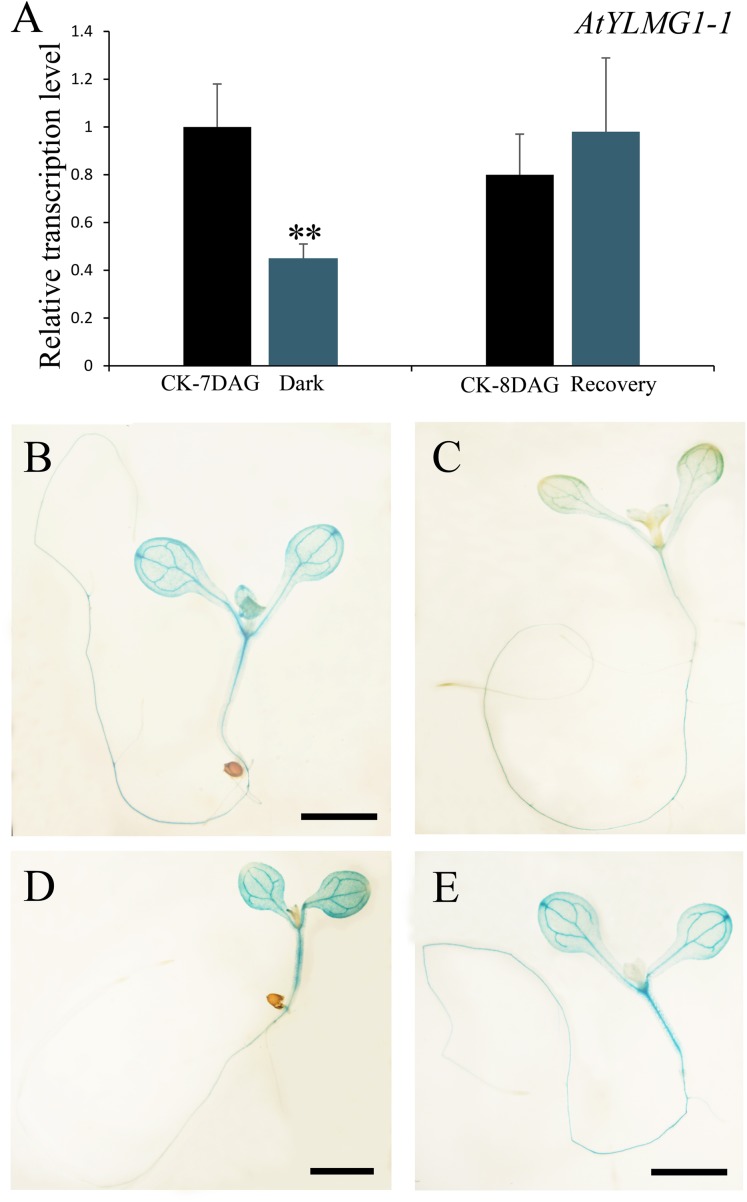
The regulation of *YLMG1-1* expression by dark and light. **(A)** Quantitative PCR analysis of *YLMG1-1* expression in seedlings. CK-7 DAG, control check of 7 DAG seedlings; Dark, 24 h dark treatment; CK-8 DAG, control check of 8 DAG seedlings; Recovery, 24 h normal light cycle after 24 h dark treatment. The vertical axis shows the relative expression levels, and the asterisk indicates a significant difference (Student’s *t*-test,^∗^*P* < 0.05, ^∗∗^*P* < 0.01). **(B–E)** GUS staining signals in seedlings from *pYLMG1-1::GUS* transgenic plants. **(B)** 7 DAG seedling in normal light cycles; **(C)** 7 DAG seedling with 6 days normal light cycles and 24 h dark treatment; **(D)** 8 DAG seedling in normal light cycles; **(E)** 8 DAG seedling under normal light cycle after 24 h dark treatment.

In higher plants, light is a necessary environmental factor for chloroplast formation (Solymosi and Schoefs, 2010). A recent study in *Arabidopsis* also revealed that differentiation from proplastid to chloroplast in embryo cells is light-dependent (Liu et al., 2017). The nuclear-encoded YLMG1-1 is targeted to the plastid and may function in chloroplast biogenesis, so we hypothesize that light can act as a regulator of *EMB1990/YLMG1-1* expression. To test this hypothesis, 7 DAG seedlings were treated under dark conditions, and then assayed by quantitative PCR to detect *YLMG1-1* transcription levels. Expression of the *EMB1990/YLMG1-1* gene was decreased by over one half in 7 DAG seedlings after 24 h dark treatment, compared to the controls (**Figure 4A**). In GUS transgenic plants subjected to the same conditions, the GUS signal was also significantly lower in dark-treated *pYLMG1-1::GUS* seedlings than in untreated controls (**Figures 4B,C**). Moreover, when the treated seedlings were removed to the normal light cycle for 24 h, expression level of *EMB1990/YLMG1-1* gene was restored to the untreated seedlings (**Figures 4A,D,E**). These results indicated that light is an important signal in regulating the expression of *EMB1990/YLMG1-1* in *Arabidopsis*.

### Chloroplast Biogenesis Is Impaired in *emb1990* Embryo Cells

**FIGURE 5:**
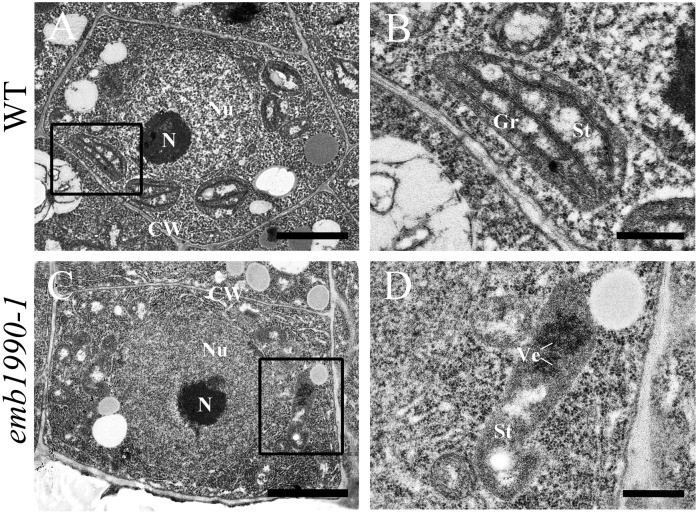
Ultrastructure observation of chloroplast in wild-type and *emb1990-1* embryo cells. **(A,B)** Cell and chloroplast in WT embryo at 5 DAP. **(C,D)** Cell and chloroplast in *emb1990-1* embryos at 5 DAP. **(B,D)** Are enlarged images of the small black boxes in **(A,C)**, respectively. N, nucleolus; Nu, nucleus; CW, cell wall; Gr, grana; St, stroma; Ve, vesicles. Scale bars = 1 μm.

Our results showed that YLMG1-1 is a plastid-targeted protein, and loss of its function disrupted embryo development after the globular stage (**Figure 2**). To clarify whether the homozygous embryo lethality was induced by dysfunction of the chloroplast in mutant embryo cells, we investigated the chloroplast structure in *emb1990* embryo cells with transmission electron microscopy (TEM). Since the albino ovules of *emb1990/*+ could not be distinguished from the wild-type ones until the normal embryo developed to the heart stage, WT and *emb1990* albino ovules from 5 DAP siliques were prepared as TEM samples for ultrastructure observation. For wild-type embryos, the cotyledon cells were selected to analyze the chloroplast structure; while the *emb1990* embryo displayed as abnormal globular shape with no formation of cotyledon primordium, the cells in entire embryo proper were observed. In the mature chloroplasts of wild-type embryo cells, thylakoid membranes had stacked into grana with well-developed lamellar organization (**Figures 5A,B**). In contrast, no granum-like structures were found in the chloroplasts of *emb1990* embryo cells, instead there were only heavily stained aggregations which indicated the membrane vesicles failed to stack and form internal thylakoid (**Figures 5C,D**). This result showed that loss function of *EMB1990/YLMG1-1* impaired the construction of thylakoid membranes in chloroplasts during embryo development, thus we concluded that the *Arabidopsis EMB1990/YLMG1-1* gene may be involved in chloroplast biogenesis, especially in the formation of thylakoid membranes.

### The Expression Levels of Certain Plastid Genes Are Affected in the Albino Ovules of *emb1990*

**FIGURE 6:**
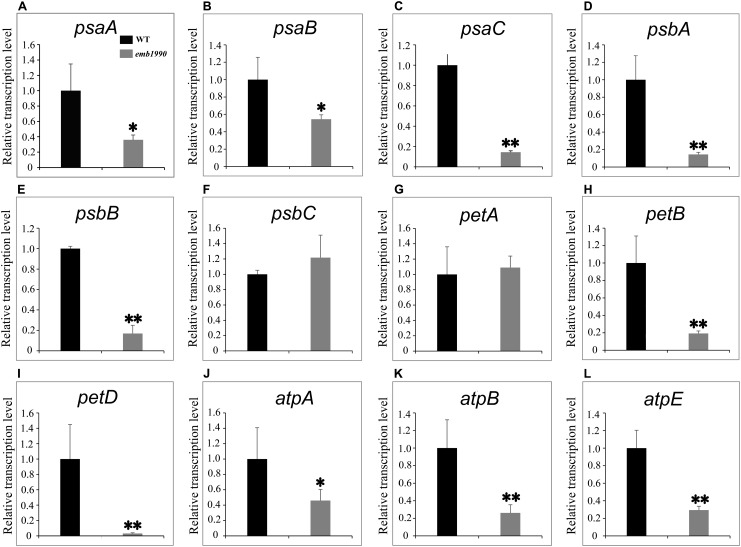
Expression analysis of plastid genes involved in thylakoid formation between WT and *emb1990-1* embryos by qRT-PCR. **(A–L)** Relative transcription level of plastid genes in WT and *emb1990-1* embryos. The RNA was isolated from normal ovules of 5 DAP in wild-type and white ovules of 5 DAP in *emb1990-1* plants. The vertical axis shows the relative expression levels, and the asterisk indicates a significant difference (Student’s *t*-test, ^∗^*P* < 0.05, ^∗∗^*P* < 0.01).

The thylakoid membrane in chloroplast is the main workplace for photosynthesis. In higher plants, the thylakoid membrane primarily contains four protein supercomplexs: photosystem I (PS I), photosystem II (PS II), cytochrome *b_6_/f* and ATP synthase, and many components of these complexes are encoded by the chloroplast genome. We found that loss of function mutations in chloroplast-localized YLMG1-1 disrupted the formation of thylakoid membranes and resulted in lethal embryos (**Figures 2**, **5**). To investigate whether the expression of chloroplast genes was influenced in the *emb1990* embryo cells, we compared the transcription levels of several chloroplast genes encoding the subunits of protein complexes in the thylakoid membrane between wild-type and *emb1990-1* embryos. The data showed that expression of 10 in the 12 examined chloroplast genes were significantly decreased in *emb1990-1* embryos compared to the wild type (**Figures 6A–E,H–L**), including *psaA*, *psaB*, and *psaC* in PS I, *psbA* and *psbB* in PS II, *petB* and *petD* in cytochrome *b_6_/f*, as well as *atpA*, *atpB* and *atpE* in ATP synthase. The down-regulation of these genes might be related to the defective construction of thylakoid membrane in *emb1990-1* embryo cells. Notably, the expression levels of *psbC* and *petA* were not affected (**Figures 6F,G**), which meant not all the chloroplast genes were impacted by the mutation of *EMB1990/YLMG1-1*. Taken together, we concluded that loss function of *EMB1990/YLMG1-1* led to affected expression of several chloroplast genes which encode the core members of protein complexes in the thylakoid membrane.

### Tissue-Specific Genes during Embryo Development Are Misexpressed in *emb1990* Embryo

Previous studies revealed that *Arabidopsis* embryogenesis was programmed by a complicated gene expression network, and several important factors were proved to function in the specification of different tissues during this precisely controlled process. In this study, we found that loss of *YLMG1-1* function led to disrupted embryos with distinct morphological defects, especially in the formation of the shoot apical meristem (SAM), cotyledon, and hypocotyl tissues. To better understand the potential mechanism of *YLMG1-1* participation in *Arabidopsis* embryogenesis, we investigated the expression levels of several important genes known to delineate the embryo cell fate decisions in *emb1990-1* albino ovules compared to the wild type.

**FIGURE 7:**
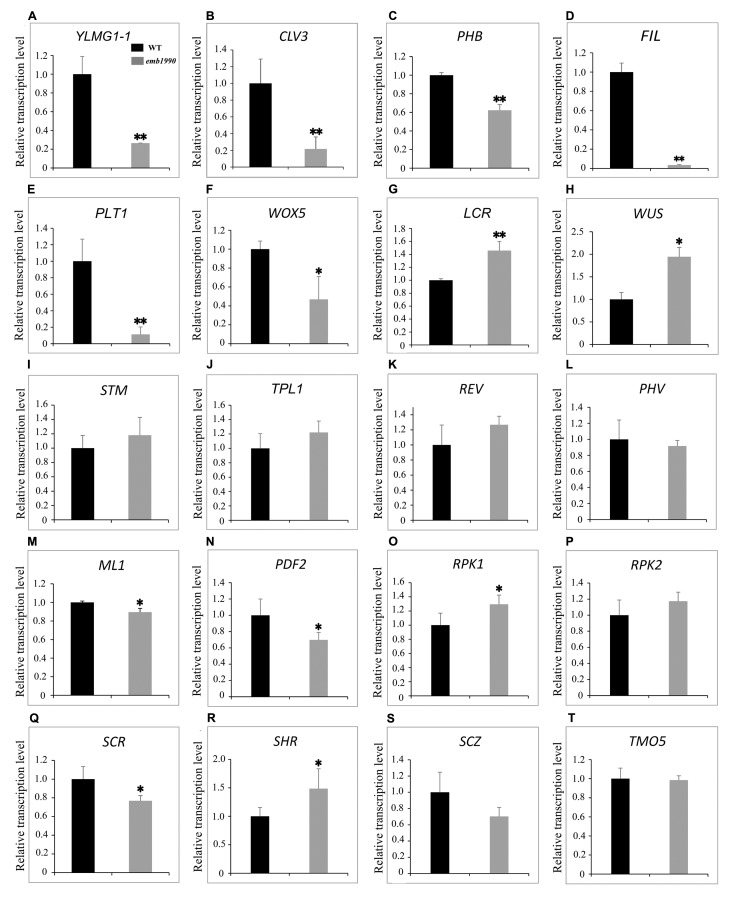
Expression analysis of nuclear genes known to delineate the embryo cell fate decisions between WT and *emb1990-1* embryos by qRT-PCR. **(A–T)** Relative transcription level of nuclear genes in WT and *emb1990-1* embryos. The RNA was isolated from normal ovules of 5 DAP in wild-type and white ovules of 5 DAP in *emb1990-1* plants. The vertical axis shows the relative expression levels, and the asterisk indicates a significant difference (Student’s *t*-test, ^∗^*P* < 0.05, ^∗∗^*P* < 0.01).

The 5 DAP ovules were used for quantitative PCR analysis. At this time the wild-type embryos had developed to the heart stage, while the abortive embryos in *emb1990-1* were arrested as abnormal globular shapes (**Figure 2**). We first confirmed the expression of *YLMG1-1* in *emb1990-1* albino ovules was decreased to only 30% of the level in wild type (**Figure 7A**), and the detected *YLMG1-1* expression might due to the seed coat used for RNA extraction. We found that most of the assayed genes were abnormally expressed in *emb1990* embryos, especially the genes that are active in regulating the formation of meristem and cotyledon. Among the extremely down-regulated genes were *CLV3* and *PHB* in SAM*, FIL* in cotyledon, and *PLT1* and *WOX5* in hypophysis (**Figures 7B–F**), all of which were corresponded to the disrupted embryo morphology in the *emb1990* mutant. Notably, not all the tissue-specific genes were down-regulated, we found that expression of *LCR* and *WUS* were increased (**Figures 7G,H**), while *STM*, *TPL*, *REV*, and *PHV* expressed in the shoot meristem were unaffected (**Figures 7I–L**), these results indicated that changed gene expression was not the consequence of developmental defects. We also found that the expression levels of some genes were less or barely influenced, such as the regulators of protoderm formation (*ML1*, *PDF2*, *RPK1*, and *RPK2*) and ground/vascular tissue (*SCR*, *SHR*, *SCZ*, and *TMO5*) (**Figures 7M–T**). Taken together, several tissue-specific genes known to delineate the cell fate decisions were misexpressed in *emb1990* embryos, suggesting that *EMB1990/YLMG1-1* plays an essential role in the embryo development in *Arabidopsis*.

## Discussion

### The Nuclear-Encoded YLMG-1 Protein Is Targeted to the Chloroplast and Required for Chloroplast Biogenesis

Chloroplast development is highly governed by nuclear genes. At the initial stage, the NEP (nucleus-encoded polymerase) and SIG (sigma factors) are imported into the proplastid to drive the expression of certain plastid genes, including the PEP (plastid-encoded polymerase) (Lopez-Juez and Pyke, 2005). The activation of PEP switches on gene transcription in the chloroplast, meanwhile numerous nucleus-encoded PPR proteins participate in RNA processing, editing and translation of plastid genes. The subsequent products synthesized in the chloroplast are assembled with other nucleus-encoded subunits to form the photosynthetic complexes (Sakamoto et al., 2008). Many nuclear genes have been characterized as necessary to maintain normal function of the chloroplast. The proteins encoded by a part of these genes are plant-specific in eukaryotes, but have homologous proteins in prokaryotes. According to endosymbiosis theory, these prokaryotic genes may be integrated into the nuclear genome from the engulfed photosynthetic cyanobacterial during the long evolution (Reyes-Prieto et al., 2007; Keeling, 2010). These nuclear genes govern the induction, transcription, and RNA processing of chloroplast genes, and represent the dominant control of cell nucleus upon the chloroplast development.

The nuclear-encoded YLMG1-1 protein of *Arabidopsis* is highly conserved within plant and bacterial taxa. In Gram-positive bacteria, *ylmG* exists in a cell division gene cluster downstream of *ftsZ*, yet inactivation of *ylmG* in *Streptococcus pneumoniae* caused no abnormalities in division (Fadda et al., 2003). Subsequent study revealed that overexpression of *YLMG1-1* perturbed the FtsZ ring formation and normal division in cyanobacteria as well as *Arabidopsis* chloroplasts, but knockdown of *AtYLMG1-1* did not affect chloroplast division (Kabeya et al., 2010). These results imply that *YLMG1-1* may have flexible function in cell/chloroplast division in different organisms. In this study, we confirmed that seed abortion in the *emb1990* mutant was caused by the deletion of *YLMG1-1* (**Figure 1** and Supplementary Figure S1). Observation of ultrastructures showed that chloroplast biogenesis was impaired in *emb1990* embryo cells, and specifically the thylakoid membrane failed to form (**Figure 5**). The thylakoid membranes of chloroplasts in *Arabidopsis* are composed of four photosynthetic complexes: PS I, PS II, cytochrome *b_6_/f*, and ATP synthase. Although many components of these complexes are encoded by the chloroplast genome, the expression of chloroplast genes was found to be primarily controlled by nuclear-encoded proteins (Kleffmann et al., 2004; Saha et al., 2007; Sakamoto et al., 2008). We also verified the expression levels of several chloroplast genes in *emb1990* embryos and found that most of the examined genes encoding subunits of protein complexes located in the thylakoid membrane were downregulated (**Figure 6**). This finding strongly suggests that nuclear-encoded EMB1990/YLMG1-1 may be required for normal expression of some important chloroplast developmental genes, and thus plays a crucial role in chloroplast biogenesis.

### YLMG-1 Participates in Embryo Development through Maintaining the Chloroplast Function

It is well known that plastids play versatile roles in plant growth and development (Inaba and Ito-Inaba, 2010). For example, many nuclear genes encoding chloroplast-localized proteins have been proved to be associated with an embryo-defective phenotype in *Arabidopsis*. Proplastid-derived chloroplast formation occurs when *Arabidopsis* embryos develop to the late globular stage. During this differentiation, the internal membrane vesicles of the proplastid are gradually linked and merged together to form thylakoid membranes, then thylakoid membranes are stacked into grana with delicate, lamellar organization (Mansfield and Briarty, 1991; Sakamoto et al., 2008). Our results indicate that the loss of function *EMB1990/YLMG1-1* mutation led to embryo development that was arrested after the late globular stage (**Figure 2**), which is likely caused by the failure of chloroplast biogenesis and misexpressed chloroplast genes (**Figures 5**, **6**). Disruption of biosynthetic functions or gene expression in the chloroplast often leads to embryo lethality, while disabling the photosynthetic machinery usually results in defective pigmentation (Bryant et al., 2011). Recently, Liu et al. (2017) showed that light deprivation-induced inhibition of chloroplast biogenesis does not arrest embryo morphogenesis, further confirming that photosynthesis and its products are not necessary for embryo development. The possibility thus remains that other various compounds synthesized in chloroplast may play essential roles in *Arabidopsis* embryogenesis, such as lipids, amino acids and phytohormones. The plastid-targeted KASI is required for the *de novo* fatty acid synthesis, and loss of *KASI* result in embryo development arrested before the globular stage (Wu and Xue, 2010). In the study of our lab, we have also found that a chaperonin subunit, CPNA2, is crucial for the transition of globular embryos to heart-shaped embryos through affecting the accumulation of KASI protein (Ke et al., 2017). These studies provided solid evidence for the important role of lipids in embryo development.

A wealth of new evidence has revealed that retrograde signaling from chloroplast to nucleus can regulate the expression of nuclear genes in response to developmental cues and stresses. Until now, over 40 components have been known to participate in the retrograde signaling, though the mechanisms or interactions for most of the components involved in this process are undetermined (Pfalz et al., 2012; Chan et al., 2016). During chloroplast biogenesis, metabolite and protein signals are sent from developing chloroplasts to the nucleus. For example, the plastidic PPR protein GUN1 is linked to the nuclear transcription factor ABI4 by the mobile chloroplast membrane-localized plant homeodomain transcription factor called PTM, which directly binds to the promoter of *ABI4* and modulates its expression (Koussevitzky et al., 2007; Sun et al., 2011). Nevertheless, the mechanism by which retrograde signaling contributes to embryogenesis remains enigmatic, and the involvement of many putative components remains unconfirmed. In the null mutant of *Arabidopsis RNase J*, disrupted embryo development was probed using fluorescent markers of specific cells, and in this case the defective chloroplast led to aberrant embryo patterning (Chen et al., 2015). In this study, we found that impaired chloroplast function caused defective embryogenesis in *emb1990* mutants, and the expression levels of several tissue-specific genes were misregulated, especially of genes known to delineate the cell fate decisions in the SAM, cotyledon and hypophysis during embryogenesis (**Figure 7**). Taken together, we propose that the nuclear-encoded YLMG1-1 protein is imported to the plastid and may be involved in maintaining the expression of certain plastid genes, subsequently the well-developed chloroplasts participate in embryo development through retrograde signaling or biosynthesis of other important compounds. Future research will focus on the mechanisms by which the impaired chloroplast may directly or indirectly alter expression of these nuclear genes in the *emb1990* mutants. These findings help us to further understand the relationship between chloroplast and embryo development.

## Author Contributions

HC analyzed the research results and wrote the paper. SL and LL performed most of the experiments and analyzed the data. HH participated in the assays of dark treatment. JZ conceived the research plans, guided the whole study, and modified the paper.

## Conflict of Interest Statement

The authors declare that the research was conducted in the absence of any commercial or financial relationships that could be construed as a potential conflict of interest.

## Footnotes

1 http://abrc.osu.edu/

2 http://suba.live/
