# Supplementary material for: Arabidopsis EMB1990 Encoding a Plastid-Targeted YlmG Protein Is Required for Chloroplast Biogenesis and Embryo Development
Source: Front Plant Sci. 2018 Feb 16;9:181. doi: 10.3389/fpls.2018.00181 (PMC5820536; doi:10.3389/fpls.2018.00181)
Supplement: Supplementary file 1 [file Table_1.PDF]

Arabidopsis EMB1990 encoding a plastid-targeted YlmG protein functions critically in chloroplast biogenesis and embryo development

Authors: Hongyu Chen†, Shuqin Li†, Lu Li†, Hengjin Hu, Jie Zhao*

Address: State Key Laboratory of Hybrid Rice, College of Life Sciences, Wuhan University, Wuhan 430072, China

***Corresponding author:** Jie Zhao
†These authors contributed equally to this work.

E-mail: jzhao@whu.edu.cn
Tel: 86-27-68756010

SUPPLEMENTARY MATERIAL

Table S1. Primers (5' to 3') used in this study.

1.1 Primers for mutant verification		
	FP	RP
emb1990-1	ccgacgggttacgattctctct	ttggggagattttggtcatgtt
emb1990-2	ccgcgaaagcaagcaatggacta	ccgcggatggttatgcagagatg
CSLB	cccatttggacgtgaatgtagacac	
1.2 Primers for complementation		
	FP	RP
EMB1900-gDNA	gcgCTGCAGtttttgtaataccaatttctac	gcgGAGCTCgagagaacgcagataatccatc
1.3 Primers for GUS/VENUS fusion constructs		
	FP	RP
EMB1900-Pro-GUS	gcgGGATCCgatttttgtaataccaatttctac	ataCTGCAGcatctccgttattccgcctgtt
EMB1900-Pro-VENUS	gcgGCATGCgatttttgtaataccaatttctac	acaGGATCCcccagtgctgccatgaacaatc
EMB1900-CDS	acgGGATCCatggccgccattacagctctca	acgGGATCCcccagtgctgccatgaacaatc

1.4 Primers for qRT-PCR		
	FP	RP
GAPDH	gagtctactggtgtcttcactg	caaggtcggacttgtattcgtg
AtYLMG1-1	tgctgagttggttcccta	gactaacatcaagcgtatcg
psaA	gggcaggacatcaagtacat	agccaaaagatcccgattca
psaB	gccaaggcttagctcaggac	cccgaaatgagaagcaaaaa
psaC	ataggatgtactcaatgtgt	tctcttacaaccaacacagt
psbA	gcgaaagcgaaagcctatgg	caatgaatgcgataataaaa
psbB	ggcgtggagggttttgac	ggcggacgaacactaagatg
psbC	acgctctttaatggaacttt	ttaatccggcatgggctaca
petA	cccgagatgaaagaaaagat	ggagcaagaataggaaaggt
petB	gatggtcggcaagtatgatgg	ggtcaatacacccagaaccaca
petD	tttcagtaccagcgggattat	gttgctccaatacctaaccaca
atpA	atggcatcgctcgtatttat	atcaaaccgtcacccattaa
atpB	acaatgctctggtggttaag	ccctctgtcgcactcatagc
atpE	ttgtgtactgactccgaatc	ttagcaaggcgtattttcaa
CLV3	aagacagccaagaaacaa	cttaccaaacgaaacaga
PHB	gcttgacgtgtggatccttct	cctttgcttccttccggtttc
FIL	cggtgcatctctcagctcctc	tgtccgatgtggttgctgtac
PLT1	gcattggacactttcggaca	ctggccttcccttctacaac
WOX5	gaggcagaaacgtcgtaaaa	aatgtctctatcaccttctc
LCR	ctctgggaaatggaaaca	tcatctgtctggacctcaa
WUS	aaccaagaccatcatctctatcatc	tcagtacctgagcttgcatga
STM	tagcctcgccacaacctc	ccttgctcctcttcctcttct
TPL1	gcgatgatattgttgtcttg	tccttatttaacatgatgac
REV	cttgtctgcgaaaatggatat	ccagcaggactattcgcatct
PHV	cagcagaataggcatcgacac	tcctctgtgccaaagcttcta
ML1	aacggaagagatgctaaagct	aacgttgccgacctattctc
PDF2	ctagcttcaacatactcttc	ggctacactcgaacgacaat
RPK1	ggagatgaagaacaccacta	ccgacagctcatcaaacca
RPK2	gggttttgcgacatttgctg	atggcaaggtcgaagatagg

SCR	ctggcttcaacattctcttta	tgcttctacaaatcttcctaa
SHR	tagccacaagatcagacgac	atccgatgcgacgccgtttg
SCZ	aaacataacaacttctctag	tctcctctcttgaaaaactc
TMO5	aagaagacgagagagaatca	tgtgatgtttgtcgttttag
