# Supplementary material for: Arabidopsis EMB1990 Encoding a Plastid-Targeted YlmG Protein Is Required for Chloroplast Biogenesis and Embryo Development
Source: Front Plant Sci. 2018 Feb 16;9:181. doi: 10.3389/fpls.2018.00181 (PMC5820536; doi:10.3389/fpls.2018.00181)
Supplement: Supplementary file 2 [file Presentation_1.pdf]

Arabidopsis EMB1990 encoding a plastid-targeted YlmG protein functions critically in chloroplast biogenesis and embryo development

Authors: Hongyu Chen†, Shuqin Li†, Lu Li†, Hengjin Hu, Jie Zhao*

Address: State Key Laboratory of Hybrid Rice, College of Life Sciences, Wuhan University, Wuhan 430072, China

***Corresponding author:** Jie Zhao
†These authors contributed equally to this work.

E-mail: jzhao@whu.edu.cn
Tel: 86-27-68756010

SUPPLEMENTARY MATERIAL

Figure S1. Genotypic analysis of *emb1990* progeny and complementation of *emb1900-2* mutant. (A, B) Genomic PCR of the T-DNA insertions in *emb1990-1* and *emb1990-2* by three primers. (C-F) Seed development in siliques of wild-type, *emb1990-2*, and complemented plants. White arrows highlight the aborted white ovules, and the siliques were placed as morphological apical to basal from left to right. Bars =1 mm.

Figure S2. Embryo development before the early globular stage in wild-type and *emb1990-1* plants. (A-D) Embryos from 2/4 cell stage to early globular stage in wild-type ovules. (E-H) *emb1990-1* embryos from siliques at different development stage as similar as wild-type embryos showed in (A-D). Scale bars = 20 μm.

Figure S3. Subcellular localization of YLMG1-1 protein in mesophyll protoplasts. (A-C) The Venus distribution in mesophyll protoplasts of *p35S::Venus* transgenic plants. (D-F) The Venus distribution in mesophyll protoplasts of *35S::AtYLMG1-1-Venus* transgenic plants. (G-I) The Venus distribution in mesophyll protoplasts of *pYLMG1-1::YLMG1-1-Venus*. Scale bars = 5 μm.
