# Supplementary figures and images for: Arabidopsis EMB1990 Encoding a Plastid-Targeted YlmG Protein Is Required for Chloroplast Biogenesis and Embryo Development
Source: Front Plant Sci. 2018 Feb 16;9:181. doi: 10.3389/fpls.2018.00181 (PMC5820536; doi:10.3389/fpls.2018.00181)

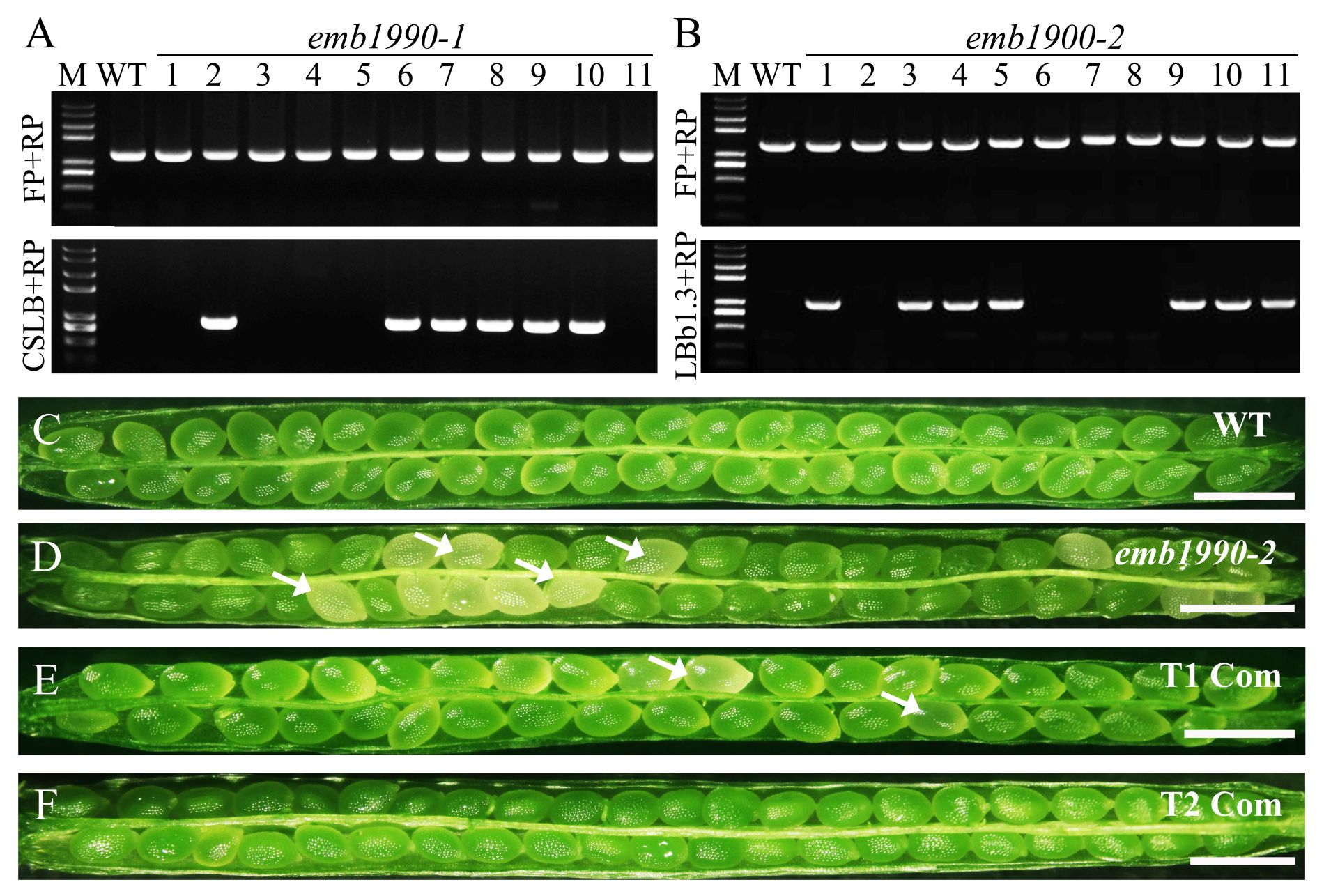

Supplement: Supplementary file 3 [file Image_1.JPEG]

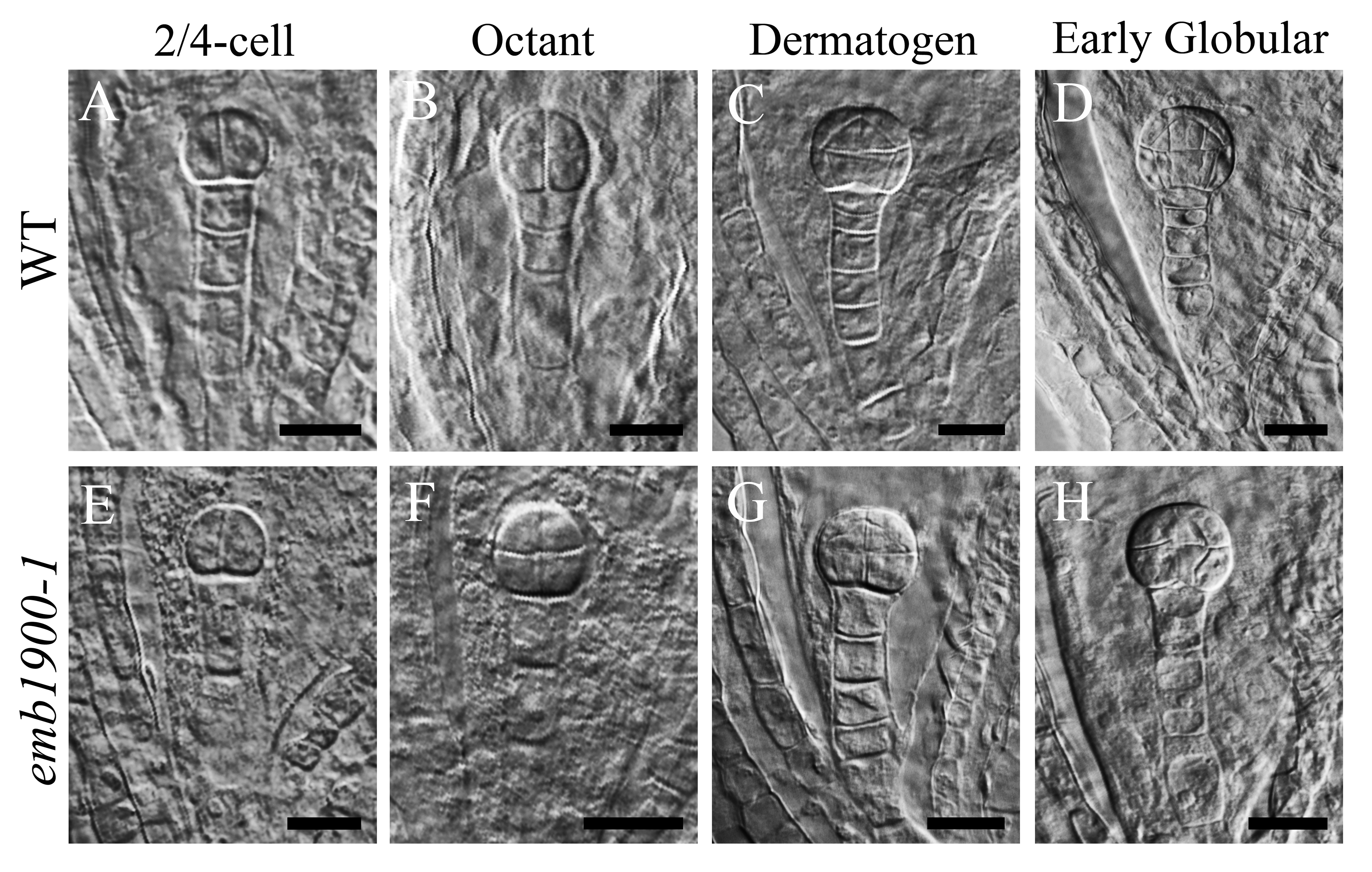

Supplement: Supplementary file 4 [file Image_2.JPEG]

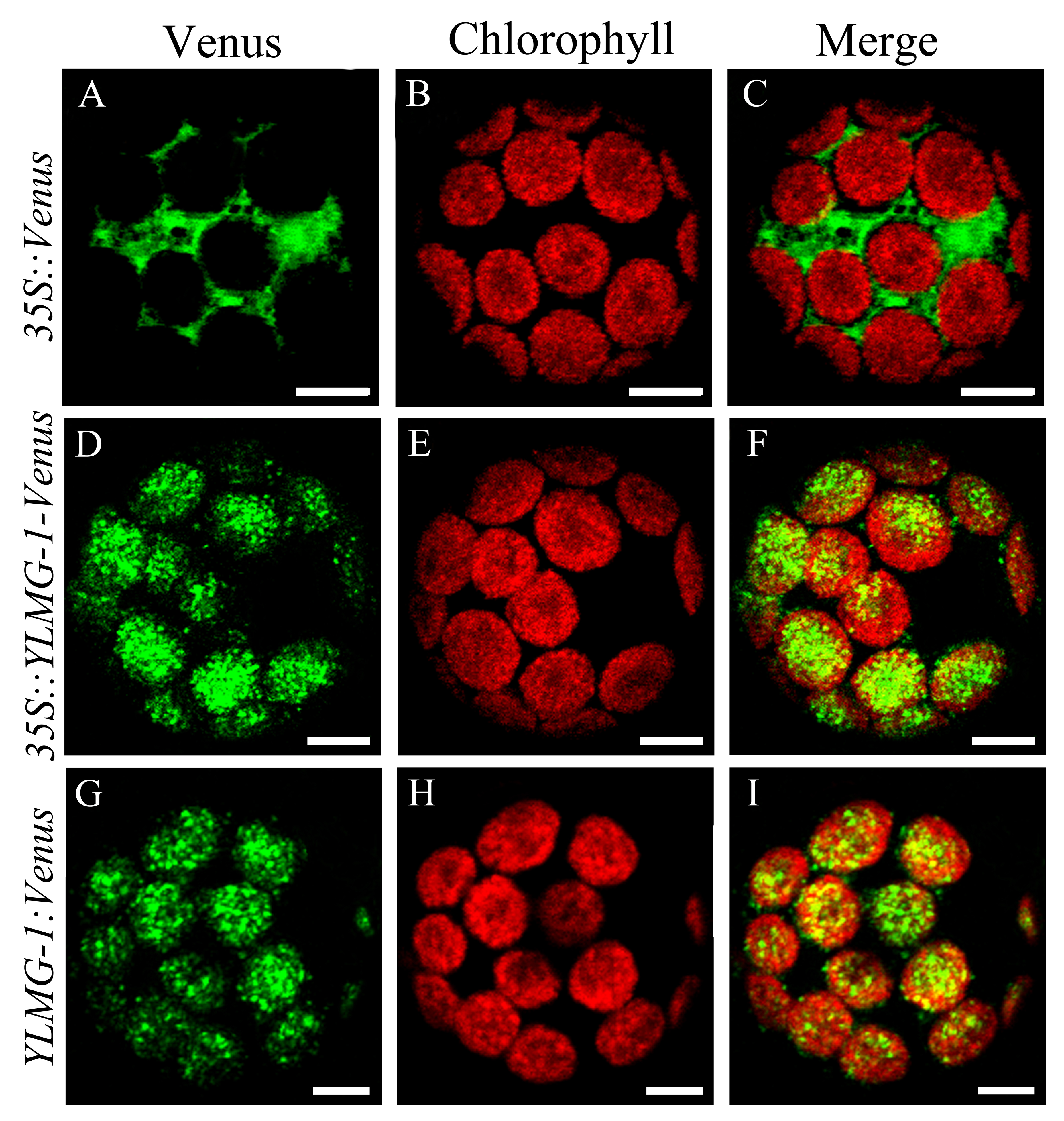

Supplement: Supplementary file 5 [file Image_3.JPEG]
